# Extra luminal colonic gastrointestinal stromal tumor: a case report

**DOI:** 10.1186/1757-1626-2-7525

**Published:** 2009-05-11

**Authors:** Ibrahim Masoodi, Mushtaq Chalkoo, Arshad Rashid, Imtiyaz A Wani

**Affiliations:** 1 Department of Medicine, Government Medical CollegeSrinagar, Kashmir 190010India; 2 Department of Surgery, Government Medical CollegeSrinagar, Kashmir 190010India

## Abstract

**Introduction:**

Gastrointestinal stromal tumors are the commonest mesenchymal tumors of the gastrointestinal tract, the stomach and small intestine are the favored sites of occurrence. They rarely occur in the colon, rectum and esophagus. GIST is neoplasm of mesenchymal origin originating from precursors of the interstitial cells of cajal. The symptoms of gastrointestinal stromal tumor depend on the site and size of the tumor, and may include abdominal pain, gastrointestinal bleeding or signs of obstruction; small tumors may, however, be asymptomatic. Majority of the patients with gastrointestinal stromal tumor have bloody stools and abdominal pain as the commonest manifestation. We describe a young female with extra luminal colonic gastrointestinal stromal tumor presenting as mass abdomen.

**Case presentation:**

We describe 34-year-old female from north Indian state of Jammu and Kashmir who had presented with history of slowly increasing epigastric lump associated with abdominal discomfort of 4 months duration. She had no features of luminal obstruction. Her contrast enhanced computed tomography abdomen revealed a large extra-colonic mass in relation to transverse colon. The tumor was resected and histology was suggestive of gastrointestinal stromal tumor.

**Conclusion:**

Extra luminal colonic gastrointestinal stromal tumors are very rare and can present as mass abdomen. Resection is the treatment of choice.

## Introduction

Colonic gastrointestinal stromal tumors (GISTs) are very uncommon. Histologically GISTs vary from spindle cell tumors to epithelioid and pleomorphic tumors. Most GISTs (95%) express Kit (CD117), CD34 (70%), and heavy caldesmon (80%), whereas 25% are positive for smooth muscle actin and less than 5% for desmin. GISTs differ histologically, immunohistochemically and genetically from leiomyomas, leiomyosarcoma and schwannomas [1]. Fine needle aspiration can be used to diagnose GISTs as spindle cell and epithelioid types, but cytomorphology alone cannot be used to assess malignant potential. Immunocytochemical staining for CD117 is helpful in confirming the diagnosis. Care must be taken to differentiate epithelioid-type GISTs from adenocarcinoma [2]. Surgery is the treatment of choice for resectable tumors. GISTs bear good prognosis after margin negative surgery. Tumor size and mitotic activity are best predictive prognostic features; small intestinal tumors behave more aggressively than gastric tumors with similar parameters [3].

## Case presentation

**Figure 1.**
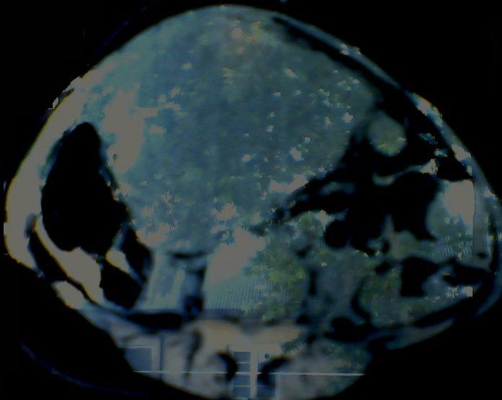
CECT abdomens showing large mass lesion in relation to transverse colon.

**Figure 2.**
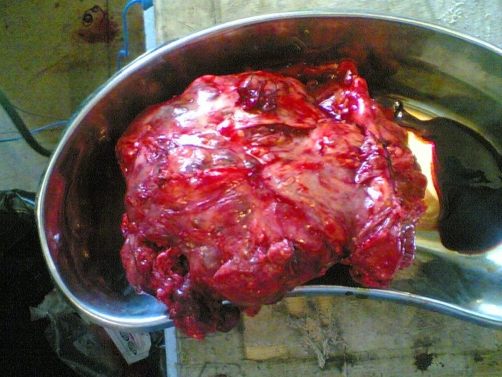
The resected tumor.

**Figure 3.**
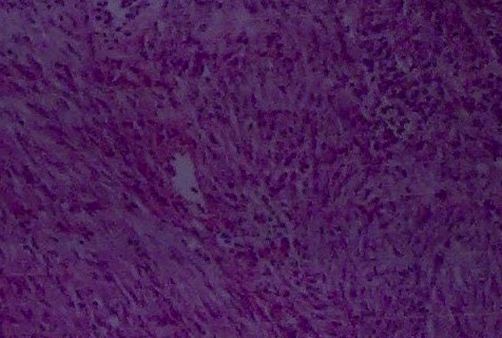
Histological picture of GIST.

A 34-year-old female from Kashmiri origin with no significant past history presented with history of awareness of abdominal lump in the epigastric region of 4 months duration. The patient had maintained appetite and no weight loss. She had no features of luminal obstruction. On examination, she was conscious, oriented with no pallor, lymphadenopathy edema or jaundice. She had a pulse of 96 beats per minute and a BP of 120/80 mmHg. Her abdominal examination revealed an abdominal lump, moving with respiration with no visible peristalsis. The swelling was 7 × 6 cms in the epigastric region, globular with smooth margins. It was firm in consistency and there was no splash or bruit on auscultation. She had no hepatosplenomegaly or free fluid. Her other systemic examination was normal. Her evaluation revealed a Hb of 11.4 gm/dl (Normal 11-14 gm/dl) TLC 10,500 (Normal 4000-10000). Her kidney function tests, liver function tests were normal. Her chest x-ray and abdominal x-ray were also normal. The contrast enhanced computed tomography (CECT) abdomen revealed 8 × 7 cms mass lesion in relation to transverse colon (Figure 1) liver, spleen etc were normal. Colonoscopy revealed extrinsic compression in transverse colon area; however, there was no luminal narrowing or intra luminal growth. Laparotomy was carried out by midline incision and growth was seen arising from the mid transverse colon along its serosal aspect. It was adherent to stomach, gallbladder and posteriorly to pancreas. Two thirds of transverse colon along with growth were removed and end to end anastomosis was done followed by loop ileostomy. Gall bladder could not be freed so cholecystectomy was also performed. Peroperative palpation of all viscera was normal. Abdomen was closed after a thorough saline wash and two drains were kept into abdomen. The patient had an uneventful postoperative course and was discharged after six days on orals. The histopathological examination of the specimen (Figure 2) revealed monomorphic eosinophilic fibrillary cytoplasm with no mitotic activity and strong immunoreactivity for KIT. The overall features were suggestive of GIST (Figure 3). She was not given postoperative chemotherapy. Patient is asymptomatic and is following our outpatient department for last 8 months now.

## Discussion

Colonic GISTs are uncommon and are usually benign. Tworek et al studied 20 colonic stromal tumors and observed that malignant ones were clinically aggressive and have metastasis at presentation causing death in short time. Authors observed that infiltrative growth pattern in the muscularis propria, invasion of the mucosa, and high mitotic counts correlated significantly both with metastases and with death from tumor [4]. GISTs usually occur as isolated lesions in colon or small bowel. However, association of small bowel GIST with synchronous colorectal cancer (CRCs) is reported. Two cases of incidental GISTs in association with CRC were reported by Melis et al.^.^The authors observed that genetic pathways of tumor genesis appeared different for the two neoplasms [5]. Although GISTs are commonly benign lesions but malignant lesions are also known. Malignant gastrointestinal stromal tumors were observed to be more common in older population, men, and blacks and in patients who never underwent surgical intervention in a series of 1458 GISTs observed over 8 years by Tran T et al [6]. Surgical resection is the treatment of choice in colonic GISTs. Abdominoperineal resection is reserved for patients with high risk or large lower rectal GISTs. Most of the workers are of the opinion that adjuvant therapy with a tyrosine kinase inhibitor Imatinib would be beneficial to high risk and lower rectal GIST patients [7]. The index case had no metastasis so only resection of the tumor was carried Imatinib 400 or 600 mg once daily was evaluated by Croom et al [8] in a randomized, nonblind multicentre study in 147 patients with advanced GIST. Authors confirmed partial responses were achieved in 54% of patients. The drug is approved for GIST and has frequent but mild side effects.

## Conclusion

We conclude that extra colonic GISTS are rare and can present as mass abdomen.

## List of abbreviations

GISTs: Gastrointestinal stromal tumors
CRC: Colorectal cancer
CECT: Contrast enhanced computerized tomography
